# Laterality Influences Agility Performance in Elite Soccer Players

**DOI:** 10.3389/fphys.2018.00807

**Published:** 2018-06-29

**Authors:** Hassane Zouhal, Abderraouf B. Abderrahman, Gregory Dupont, Pablo Truptin, Régis Le Bris, Erwan Le Postec, Sullivan Coppalle, Guillaume Ravé, Matt Brughelli, Benoit Bideau

**Affiliations:** ^1^Movement Sport Science and Health Laboratory (M2S), UFR-STAPS, University of Rennes 2 – ENS-Rennes, Bruz, France; ^2^ISSEP Ksar Said, University of Manouba, Tunis, Tunisia; ^3^Fédération Française de Football (FFF), Paris, France; ^4^Lorient Football Club (FCL) and Stade Rennais Football Club (SRFC), Lorient, France; ^5^Stade Lavallois Mayenne Football Club, Laval, France; ^6^Sports Performance Research Institute New Zealand (SPRINZ), AUT Millennium, Auckland University of Technology, Auckland, New Zealand

**Keywords:** laterality, football, footedness, eyedness, rotation

## Abstract

**Introduction:** Laterality (i.e., handedness, footedness, and eyedness) could have an impact on highly repeated soccer movements and thus, could influence performance. The purpose of this study was to examine the laterality of high-level football players and its effects on 180° left and right U-turn movements.

**Materials and Methods:** Handedness, footedness, and eyedness were determined in 72 elite football players (EFP, 18.2 ± 2.2 years) from the Stade Rennais Football Club (French League 1) and 9 amateur football players (AFP, 19.6 ± 2.1 years). Players performed a visual-motor task on a synthetic pitch consisting of 180° left and right rotations as fast as possible in response to a visual light on a computer screen. Movement times and reactive times for each left and right rotation were recorded with an accelerometer and video display.

**Results:** Laterality profiles showed a majority (χ^2^ = 9.42, df = 2, *p* = 0.031) of crossed formulas (i.e., dominant leg or hand is controlateral to the dominant eye) for EFP (53 ± 7%) and a majority of non-crossed formulas for AFP (63 ± 9%). Reaction times were significantly faster (*p* = 0.028, effect size = 0.148, trivial) in EFP right-eyed (568.2 ± 55.5 ms) than in AFP (610.0 ± 43.9 ms). For the left rotation and for right-footed players, movement times were significantly different (*p* = 0.043, effect size = 0.413, small) between EFP (1.15 ± 0.07 s) and AFP (1.17 ± 0.07 s). A significant difference (*p* < 0.033) was observed between footedness and rotation movement times in the EFP.

**Conclusion:** Our results showed that laterality profiles differed between EFP and AFP. Hence, in EFP, reaction times depended on the side of the visual stimulus. Moreover, leg laterality of EFP influenced 180° left or right rotation speed. Our results indicate the importance of determining laterality in soccer players and identifying deficits in performance when turning.

## Introduction

Soccer is a team sport characterized by highly repeated multidirectional movements (Little and Williams, 2005; Bloomfield et al., 2007a). The ability to react quickly and effectively while executing these movements is vital for performance, and is often referred to as agility (Young et al., 2002; Sheppard and Young, 2006). Sheppard and Young have defined agility as ‘a rapid whole body movement with change of velocity or direction in response to a stimulus’ (Sheppard and Young, 2006). Agility has two main components: change of direction ability, and perceptual and decision making ability (Young et al., 2002). The physical determinants of agility have been investigated by several authors (Young et al., 2002; Little and Williams, 2005; Sheppard and Young, 2006; Barnes et al., 2007; Brughelli et al., 2008; Chaouachi et al., 2012) but many of them only focused on change of direction ability (Young et al., 2002; Sheppard and Young, 2006). Agility performance has not been strongly linked with straight sprint speed (Little and Williams, 2005; Sheppard and Young, 2006; Brughelli et al., 2008; Chaouachi et al., 2012) nor with leg muscle strength or power (Young et al., 2002; Sheppard and Young, 2006). However, agility performance has significantly correlated with running techniques, height, relative limb lengths, and the height of the athlete’s center of gravity (Sheppard and Young, 2006).

(Sheppard and Young, 2006; Sasaki et al., 2011), and reactive strength (Sheppard and Young, 2006). Moreover, agility has been highlighted as a good parameter for talent identification in soccer (Reilly et al., 2000). Recently, Slimani and Nikolaidis (2017), in their systematic review identified agility as a key prerequisite for elite players compared to all other competitive levels (i.e., sub-elite, amateur, recreational).

A previous study found that athletes who changed directions faster to one side tended to have reactive strength dominance in the leg responsible for the push-off action (Young et al., 2002). Hence, it has been demonstrated that reactive strength and motor control were better in the support leg, which was not the dominant one (Carey et al., 2001; Valdez, 2003). In soccer, several studies showed that preferred leg kicks were somewhat faster (or somewhat more accurate), but the advantages were not substantial. Other researchers have investigated if strength advantages exist in the dominant leg, but the effects obtained have usually been small and unrelated to performance in kicking (Kramer and Balsor, 1990; McLean and Tumilty, 1993; Mognoni et al., 1994).

Moreover, in soccer, rotation movement has been established as a major determinant of match performance in elite soccer (Bloomfield et al., 2007a). It has recently been reported that within a match, players of different positions performed more than 700 rotations and turns (Withers et al., 1982; Bloomfield et al., 2007a,b). Among these rotations, more than 300 rotations were performed from 0 to 180° (Withers et al., 1982; Bloomfield et al., 2007a,b). This could be explained by the efforts in close encounters to evade a marker, or aspects of match-play where players are required to face their own goal and the ball is transferred overhead (e.g., goal-kick) (Bloomfield et al., 2007a). So one can assume that footedness may have an influence on rotation performance (e.g., change of direction component of agility).

Concerning laterality, some authors have assessed handedness, footedness and eyedness of a range of athletes from different sports, thus establishing a database of laterality (i.e., laterality profile) (Brullebaut and Gillot, 1981; Bisiacchi et al., 1985; Azemar, 1998; Azémar, 2003; Sommer, 2006; Azemar et al., 2008; Loffing et al., 2010). However, to the best of our knowledge, only one study included soccer players and the authors only assessed footedness during soccer matches (Sommer, 2006). On the other hand, in various sports the dominant eye allowed players to capture and treat information faster in his/her visual field (Azémar, 2003; Mapp et al., 2003; Azemar et al., 2008). Then a question arises concerning if eyedness might influence the reaction time to a visual stimulus (e.g., the perceptual and decision making component of agility). Hence, determination of the dominant side may help coaches to choose the playing position for their players especially for specific positions as central and lateral defenders. Consequently, the first purpose of this study was to observe the impact of laterality on agility in soccer players. The second purpose was to assess the distribution of eyedness, handedness and footedness of a professional high-level soccer population. We hypothesize that (i) laterality would affect soccer performance during a single direction change; and (ii) soccer players would have an over-representation of lateral crossed formulas (e.g., dominant eye and dominant hand opposite) as in fencing and tennis (Azémar, 2003; Azemar et al., 2008).

The common agility tests presented in the literature usually only measure change of direction ability (CODA), without a response to stimulus. According to several authors (Sheppard and Young, 2006; Sasaki et al., 2011; Pojskic et al., 2018), a response to a visual stimulus must be included to efficiently test agility in soccer players. Thus, we modified an agility test with a single direction change, based on Sheppard and Young (2006; Sasaki et al., 2011), which included a response to a visual stimulus.

## Materials and Methods

### Participants

Seventy-two male elite football players (EFP) from the Stade Rennais Football Club (French Ligue 1) aged 15–30 years, median = 18.1 years, and nine male amateur football players (AFP) aged 17–25 years, median = 20.2 years, participated to this study. All participants were injury free at the time of testing. Among EFP, several teams were enrolled: Under 16 (U16), Under 17 (U17), Under 19 (U19), the reserve team and the professional team. EFP were contrasted with AFP. Before the study, written informed consent was obtained from each participant and their parents or guardians in accordance with the international ethical standards and the study was approved by the Ethical Committee on Human Research of the University of Rennes 2, France, and was conducted according to the Declaration of Helsinki and its later amendments.

### Procedures

Firstly, all the players completed a sport related questionnaire to determine their laterality profile. Among the laterality assessment questionnaires available in the literature, the one of Azémar (2003) was chosen. It is in French, and is a valid and reliable test, which reflects the dynamic and coordinated, set of lateral specialization during athletic movements. The items refer to sport specific situations, for example: *which hand do you use to throw a ball* or *which foot do you use to kick a ball*. For each items the participants had to tick R for right and L for left. The assessment of eyedness, handedness, and footedness were obtained which established the athletes’ laterality profile.

**FIGURE 1:**
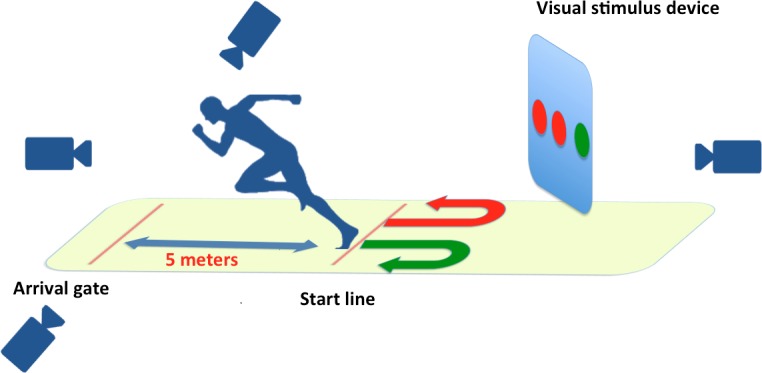
Equipment set up for the agility test. The device showed the side of the rotation: when the left light turned red, the player performed a left rotation and conversely when the right light turned red, the player performed a right rotation. When the center light turned red, the player chose the preferable side to turn. The device was seated on a tripod with adjustable height, therefore each test was arranged at the player’s eye level. To achieve the agility test, the player had to execute a 180° turn and run as fast as possible to the arrival gate placed 5 m behind him.

Secondly, 15 EFP (14 U19 and 1 from the reserve team) and nine AFP (**Table 1**) performed a modified agility test (**Figure 1**). According to the general information provided by Barnes et al. (2007) the specific CODA in the current study was represented by only one change of direction (i.e., 180° turn to the right or to the left, depending on visual stimulus) and sprinting for 5 m. In fact, these authors Barnes et al. (2007) reported that in team-sports, players were mostly subjected to sharp change of directions (i.e., 180° turn) during sprints of approximately 5 m. Consequently, change of direction tests involving these characteristics would likely be ideal for specific CODA assessments in team-sports. Specifically, in competitive soccer, functional shuttle running takes the form of very short sprints supporting the relevance for soccer-specific CODA assessment (Bloomfield et al., 2007b; Chaouachi et al., 2012). Among the agility tests in the literature, none fit with our specific movement to study 180° rotation. Thus we create a test taking into account the definition of agility as ‘sprints with directional changes in response to a stimulus’ (Sheppard and Young, 2006). The test consisted of a 180° rotation in response to a visual stimulus which indicated the side of the rotation (**Figure 1**). The player started in standing position, with parallel feet. Two meters in front of the athlete was an electronic device, which displayed the visual stimulus, and five meters behind him was an arrival gate. The device included a screen that captured data from a computer. Application software Virtools generated real-time 3D visual lights (Virtools Software Suite 3.5, Copyright© 2006 Dassault Systemes, France). The lights were composed of three balls of green colors modeled in 3D. Each ball had the same size and lighted with the same intensity. The device showed the side of the rotation: when the left light turned red, the player performed a left rotation and conversely when the right light turned red, the player performed a right rotation. When the center light turned red, the player chose the preferable side to turn. The device was seated on a tripod with adjustable height, therefore each test was arranged at the player’s eye level. To achieve the agility test, the player had to execute a 180° turn and run as fast as possible to the arrival gate placed 5 m behind him. Each player had to execute the test nine times: three times for each light in randomized order due to the software: left, right, or center rotation. Before the test each player performed a 20 min standardized warm-up and 1 min of recovery was allowed between each trial. After the initial familiarization session and in order to assess test–retest reliability, all players performed the test two times on two different days separated by at least 2 days. The testing procedure and time of day were identical for all players. Players were asked to not train 24 h prior to testing to minimize the effects of fatigue. Testing took place on artificial turf, and the environmental conditions such as temperature and wind were recorded and were similar between the two testing sessions. Players were instructed to wear the same footwear for all sessions.

**Table 1:** Player’s data for the agility test.

	*n*	Age (year)	Height (cm)	Body mass (kg)	Body fat (%)
**EFP**	15	18.5 (0.8)	179.0 (5.1)	68.9 (6.9)	11.1 (1.5)^∗^
**AFP**	9	19.6 (2.1)	178.0 (4.8)	66.3 (4.6)	13.6 (1.1)
**Total**	**24**	**19.0 (1.4)**	**178.5 (4.9)**	**67.6 (5.7)**	**12.3 (1.3)**

*Data are means (±SD).*

*EFP, elite football players; AFP, amateur football players.*

*^∗^Significantly different between EFP and AFP, p < 0.05.*

### Collecting Data

The player’s reaction times (RT) were obtained with an accelerometer, disposed at the chest height and connected via Bluetooth to a computer. The time between the signal appearance and the beginning of player movement was recorded. The player’s movement times (MT) were collected by four cameras SANYO HD (Sanyo VCB-3170P CCD CamerA B/W, Japan) placed all around the test area (see **Figure 1**). The measures were recorded with a frequency of 50 Hz and calculated with Kinovea software (Kinovea^®^ software rel 0.8.7, France1,^1^).

### Statistical Analysis

Results are expressed as means ± standard deviations (SD). Statistical analysis was performed with SPSS for Windows, version 16.0 (SPSS Inc., Chicago). All variables used in the study were checked for normality of distribution before the analyses (Shapiro–Wilk test). On the basis of a power analysis (expected SD of residuals = 50 ms for RT and 0.05 s for MT, desired power = 0.80, and alpha error = 0.05), we determined that a sample size of *n* = 7 per group would be sufficient to detect differences between the two groups. The reliability of the test was assessed by intra-class correlations (ICCs). For assessing intra- and inter-rater reliability, ICC agreement values with 95% CI were calculated (De Vet et al., 2006). ICC agreement was preferred as it takes systematic and random errors into account (De Vet et al., 2006). χ^2^-tests were used to define possible differences in distribution of handedness, footedness, and eyedness. Independent *t*-tests were used to compare laterality profiles of AFP versus EFP for MT and RT means. Moreover, analysis of variance (ANOVA) was used for comparison between group factors (i.e., right-footed/left-footed). Practical differences in performances between AFP and EFP were assessed by calculating the Cohen’s *d* effect size (Cohen, 1988). Effect sizes (ES) between <0.2, 0.2–0.6, 0.6–1.2, 1.2–2, and 2.0–4.0 were considered as trivial, small, moderate, large and very large, respectively (Hopkins et al., 2009). Significant differences were assumed when *p* < 0.05.

## Results

### Reliability of the Test

The ICCs for reaction times and movement times measured over the two testing sessions using paired sample *t*-tests revealed no significant differences between the two testing occasions. The test–retest [intra-class correlation coefficient (ICC)] analysis revealed a high level of reliability between the two testing sessions (*r* = 0.904 for RT and *r* = 0.898 for MT). For this test, the intra-rater reliability ranged from moderate to almost perfect agreement (ICC ≥ 0.48–0.82) according to the classification of Landis and Koch (1977).

### Athletes’ Laterality

**FIGURE 2:**
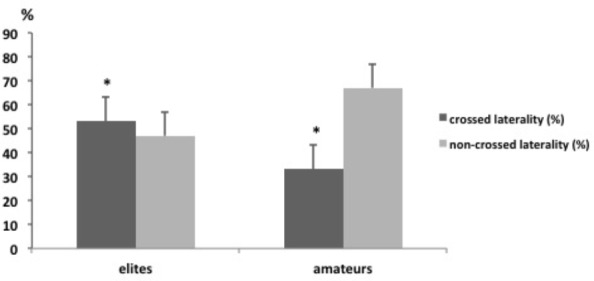
Crossed laterality (e.g., dominant hand or/and dominant foot opposite to the dominant eye), of elites and amateurs players. ^∗^Significantly different between elites and amateurs, *p* < 0.05.

Descriptive data of laterality profiles can be found in **Table 2**. It can be observed that the most represented profile is the right eyed, right handed, and right footed one, with an average of 66, 92, and 82%, respectively. Moreover, for both AFP and EFP, it was found that the majority of players had a of right side preference for the distribution of handedness, eyedness and footedness. However, in EFP it appeared that eyedness had the strongest percentage of left-preference with an average of 36% vs. 22% compared to AFP. As presented in **Figure 2**, the percentage of crossed-laterality (e.g., dominant hand or/and dominant foot opposite to the dominant eye) was significantly higher (χ^2^ = 9.38, df = 2, *p* = 0.031) in elite players (53%) compared to amateur ones (33%).

**Table 2:** Distribution of eyedness, handedness and footedness within all participating groups, as assessed by the Azemar’s questionnaire measuring functional preference for different sport skills.

Sample	*n*=	Eyedness		Handedness		Footedness	
				
		Left (%)	Right (%)	Left (%)	Right (%)	Left (%)	Right (%)
U16	8	3 (37)	5 (63)	0 (0)	8 (100)	3 (37)	5 (63)
U17	17	8 (47)	9 (53)	1 (5)	16 (95)	2 (11)	15 (89)
U19	14	5 (35)	9 (65)	3 (21)	11 (79)	3 (21)	11 (79)
Reserve team	12	3 (25)	9 (75)	2 (16)	10 (84)	3 (25)	9 (75)
Professional team	21	7 (33)	14 (67)	0 (0)	21 (100)	3 (14)	18 (86)
**Total EFP**	**72**	**26 (36)**	**46 (64)^∗^**	**6 (8)**	**66 (92)^∗^**	**15 (20)**	**58 (80)^∗^**
AFP	9	2 (22)	7 (78)	1 (11)	8 (89)	1 (11)	8 (89)
**Total (EFP + AFP)**	**81**	**28 (34)**	**53 (66)**^∗^	**7 (8)**	**74 (92)**^∗^	**15 (18)**	**66 (82)**^∗^

*Data are means (±SD).*

*EFP, elite football players; AFP, amateur football players.*

*^∗^Significantly different between Left and Right, p < 0.05.*

**Table 3** shows the distribution of eyedness, handedness, and footedness in elite football players according to playing position. It can be observed that, except for goalkeepers, the most represented profile was right eyed, right handed, and right footed for defenders, midfielders, and forward. However, for goalkeepers it appeared that eyedness had the strongest percentage of left-preference compared to other players with an average of 63% vs. 27, 38, and 41%, respectively, for defenders, midfielders and forward.

**Table 3:** Distribution of eyedness, handedness, and footedness in elite football players according to playing positions.

Sample	*n*=	Eyedness		Handedness		Footedness	
				
		Left (%)	Right (%)	Left (%)	Right (%)	Left (%)	Right (%)
Goalkeepers	8	5 (63)**^∗^**	3 (37)	1 (12)	7 (88)**^∗^**	0 (0)	8 (100)**^∗^**
Defenders	26	7 (27)	19 (73)**^∗^**	2 (8)	24 (92)**^∗^**	4 (15)	22 (85)**^∗^**
Midfielders	26	10 (38)	16 (62)**^∗^**	6 (23)	20 (77)**^∗^**	8 (30)	18 (70)**^∗^**
Forward	12	5 (41)	7 (59)^∗^	2 (16)	10 (84)^∗^	3 (25)	9 (75)^∗^

*Data are means (±SD).*

*^∗^Significantly different between Left and Right, p < 0.05.*

### Athletes’ Performances

As shown in **Table 4**, among EFP, the RT of the right-eyed players was significantly lower (*p* < 0.028, effect size = 0.141) than the left-eyed. Hence, the right-eyed players reacted significantly faster (*p* = 0.025, effect size = 0.118, trivial) when the stimulus appeared on the right side, and conversely the left-eyed players reacted significantly faster (*p* = 0.031, effect size = 0.335, small) when the stimulus appeared on the left side. However, no significant difference was observed among AFP between the RT and the side of the stimulus whether the amateur players were left eyed or right eyed. Hence, the right-eyed EFP reacted significantly faster (*p* = 0.028, effect size = 0.118, trivial) when the stimulus appeared on the right side compared with the AFP.

**Table 4:** Athletes’ performance on agility test: reaction times.

Side of the visual stimulus	AFP RT (ms)		EFP RT (ms)		Repeated measures (*P*)	Effect size
				
Effect	Left-eyed	Right-eyed	Left-eyed	Right-eyed		
**Right side**	612.8 (0.7)	604.5 (53.9)$	677.0 (65.9)^∗^£	548.6 (56.7)^∗^£$	0.450	0.118
**Left side**	599.3 (17.9)	615.3 (34.8)	587.9 (49.3)^∗^£	638.8 (84.1)^∗^£	0.173	0.335
**Total**	**606.0 (12.9)**	**610.0 (43.9)$**	**658.0 (74.0)^∗^**	**568.2 (55.5)^∗^$**	**0.185**	**0.141**

*Data are means (±SD).*

*AFP RT, amateur football player’s reaction time; EFP RT, elite football player’s reaction time.*

*^∗^Significantly difference between right-eyed and left-eyed players. ^∗^p < 0.05.*

*^£^Significantly difference between right side and left side visual stimulus. ^£^p < 0.05.*

*^$^Significantly difference between EFP and AFP. ^$^p < 0.05.*

Concerning the performance on the agility test, results showed no significant differences between EFP and AFP concerning movement times (**Table 5**). However, in both EFP and AFP, MT was significantly lower (*p* = 0.043, effect size = 0.413, small) when the players turned on the controlateral side to their dominant foot. The right-footed players were faster on the left rotation and conversely the left-footed players were faster on the right rotation. Significant differences (*p* = 0.039, effect size = 0.413, small) were observed for MT between right and left footed players in EFP for both left and right rotation and only for the right rotation for AFP.

**Table 5:** Athletes’ performances on agility test: movement times.

Side of the rotation	AFP MT (s)		EFP MT (s)		Repeated measures (*P*)	Effect size
		
	Left footed	Right footed	Left footed	Right footed		
Right rotation	1.13 (0.08)£	1.22 (0.07)^∗^£	1.11 (0.01)^∗^£	1.20 (0.02)^∗^£	0.013^∗^	0.974
Left rotation	1.16 (0.02)£	1.17 (0.07)£	1.18 (0.06)^∗^£	1.15 (0.07)^∗^£	0.357	0.413
**Total**	**1.15 (0.06)**	**1.19 (0.06)**	**1.15 (0.05)**	**1.17 (0.06)**	**0.452**	**0.097**

*Data are means (±SD).*

*AFP MT, amateur football player’s movement time; EFP MT, elite football player’s movement time.*

*^∗^ Significantly difference between right-footed and left-footed players. ^∗^p < 0.05.*

*^£^Significantly difference between right side and left side visual stimulus. ^£^p < 0.05.*

## Discussion

The present study was the first to examine laterality of high-level football players and its effects on performing 180° left and right rotations. The influence of laterality on a U-turn movement, with a special reference to leg dominance, was studied and the performance was compared between high level and amateur football players. The main findings showed a majority (*p* < 0.05) of crossed formulas (e.g., dominant leg or hand is controlateral to the dominant eye) for EFP and a majority of non-crossed formulas for AFP. Moreover, the reaction times depended on the side of the stimulus and EFP reaction times were faster than AFP. Hence, the results demonstrated that the movement times depended on the side of the rotation and appeared to be faster in both EFP and AFP when the player turned on the controlateral side with their dominant foot.

### Results of Questionnaire

Concerning football player laterality, the same distribution can be observed in the general population. Regarding eyedness, it can be observed that 66% of the players were right-eyed. Except for goalkeepers, similar results were observed for defenders, midfielders and forward. Our data were consistent with the literature: Sommer (2006) found an average of 69%. Moreover, only 8% of the players were left handed, whereas in the general population an average of 9–10% can be observed. Concerning footedness, 18% were left footed, and 82% right footed; the same distribution can be observed in the general population (Sommer, 2006). Similar results were observed according to player position. But it is important to consider the fact that high level soccer players are also skilled, on average, with their non-preferred foot, (Sommer, 2006) therefore it could be useful to add ‘mixed footed’ in the questionnaire because some of the players in the current study didn’t really know if they were more skilled with their right or left foot. When in doubt, they generally chose their right foot. However, these results concern only football players and it is well known that laterality profiles differ among sports (Azémar, 2003). Nevertheless, to the best of our knowledge, this was the first study to record the distribution of eyedness, handedness, and footedness among high-level football players. Concerning cross laterality, results from the current study showed a majority of crossed formulas (e.g., dominant leg is controlateral to the dominant eye) for EFP. Thus, more studies are needed to demonstrate if soccer allows specific laterality profiles for performing at the highest-level.

### The Agility Test

To the best of our knowledge, there has been no previous research on reliability values for an agility test, which included a response to a visual stimulus and a single direction change. The test–retest ICC values for this new test were in the same range as some previous research studies involving tests of planned direction changes (Alricsson et al., 2001; Pandorf et al., 2003; Sheppard et al., 2006) and higher than others that measured the reliability involving unplanned direction changes (Chelladurai et al., 1977; Hertel et al., 1999; Farrow et al., 2005). Importantly, all of the tests produced ICC values that were acceptably reliable (>0.80) for physical performance tests (Thomas and Nelson, 2001; Sheppard et al., 2006).

Consequently, the lack of a significant difference between the test results, along with the high ICC values, indicates that the test used in the current study is acceptable and test–retest reliable. For the reaction times, better results were obtained by the EFP. This could be explained by better information-perception coupling compared to the AFP (Vaeyens et al., 2007). Among EFP, the dominant right-eyed players had significantly better reaction times than the dominant left eyed, *p* < 0.05 (**Table 4**). Plus, the dominant right-eyed players reacted faster when the stimulus appeared on the right side, and conversely the left eyed players reacted faster when the stimulus appeared on the left side. However, we found no significant differences among AFP players between the reaction times and the side of the stimulus whether they were left eyed or right eyed. Additionally, the EFP were found to have greater reaction times when the signal appeared on the side of their dominant eye. These results can be explained by the role of the dominant eye in the information treatment. When the stimulus appeared in the dominant eye’s visual field, it was captured and treated by the visual cortex in priority and thus caused a faster motor response (Mapp et al., 2003). On the contrary, if the stimulus appeared far from the dominant eye field, it would not be treated in priority and so the motor response would be slower. Hence, no significant differences were observed among the AFP for reaction times. This could be due to the small number of players.

Several studies have indicated that agility is a performance factor, which differentiates elites and amateur players (Sasaki et al., 2011). Recently, Slimani and Nikolaidis (2017) demonstrated in their systematic review that higher VO2max, muscle strength, muscular power (vertical jump height), running speed (10–30 m) and agility, and lower % of body fat were identified as key prerequisites in elite players compared to all other competitive levels (i.e., sub-elite, amateur, recreational). These findings were not confirmed by our results concerning movement times. In fact, EFP were not significantly faster than the AFP for movement times. However, the left footers were faster when they performed a right rotation and conversely, the right footers were fasters when they performed a left rotation. This result means that the players were better when they turned with their supporting leg. For example, when a right-footed player turned to the left he used his supporting leg, but when he turned to the right he used his dominant leg. The supporting foot is the one that is not used to shoot the ball (Sommer, 2006). The players were faster when they turned with their supporting leg because they had more strength and better motor control (Valdez, 2003). Consequently, movement time were not found to be significantly different between AFP and EFP on the agility test. Change of direction speed during a 180° rotation with a standing start wasn’t related with player level. However, some reliable and valid studies have found that change of direction speed and other movements were correlated with playing level (Reilly et al., 2000). In our study, this ability cannot be taken as talent identification. A focus on training this ability is needed.

A limitation should be acknowledged in the current study. In fact, only nine amateur players participated to the experiment which may affects the findings in term of reliability of data concerning handedness, footedness, and eyedness differences.

### Practical Applications

Agility is known to be important for soccer performance, and laterality seems to influence agility. The current study provides results concerning laterality and its influence on agility. The findings, in our opinion, suggest that head coaches, fitness coaches and staff members working with soccer players may need to more carefully:

• Determine and analyze laterality (i.e., handedness, footedness and eyedness) of their players. This may help to choose accurately the playing position of the players according to their dominant side (especially eyedness). For example, for the two central defenders, the one of them who has left eydeness could be placed at the left of the other central defender.
• Measure and analyze RT and MT in response to a standardized stimulus. This may help to detect the weak and the strong sides of each player.
• Train players to increase their ability to turn (weak side vs. strong side).

## Conclusion

The current study showed that laterality of elite soccer players’ was similar with the general population, and that a majority of crossed formula was observed in EFP. Thus it seems that soccer does not allow specific laterality profiles at the highest level, but crossed formula may be an indicator. Moreover, laterality had an impact on the players’ agility. The dominant eye permits a priority treatment of the information on its visual field and thus allows faster reaction times. The supporting leg permits more reactive strength and a better motor control of push-off actions, thus allows turning faster on the opposite side. Therefore each player had a weak side and a strong side when he performed a 180° rotation, which is why this ability must be trained.

## Author Contributions

HZ conceived and designed the research. PT, RLB, ELP, SC, BB, and GR conducted the experiments. PT and AA analyzed the data. PT, GD, MB, AA, BB, and HZ wrote the manuscript. All authors read and approved the manuscript.

## Conflict of Interest Statement

The authors declare that the research was conducted in the absence of any commercial or financial relationships that could be construed as a potential conflict of interest.

## Footnotes

1 https://www.kinovea.org/
